# Engineered Microenvironments for 3D Cell Culture and Regenerative Medicine: Challenges, Advances, and Trends

**DOI:** 10.3390/bioengineering10010017

**Published:** 2022-12-22

**Authors:** Anna Guller, Alexandra Igrunkova

**Affiliations:** 1 Macquarie Medical School, Macquarie University, Sydney, NSW 2109, Australia; 2 World-Class Research Centre “Digital Biodesign and Personalized Healthcare”, Sechenov First Moscow State Medical University, Moscow 119992, Russia

The overall goal of regenerative medicine is to restore the functional performance of the tissues and organs that have been severely damaged or lost due to traumas and diseases. This goal may be achieved by stimulating the body’s regenerative capacities, or by replacing the affected anatomical structures with the missing tissue components. The primary methodology addressing this challenge is known as tissue engineering. As an engineering approach, it implies finding reliable solutions via rational design of the new systems from the defined components. The conventional tissue engineering triad of essential components includes cells, scaffolds (as mimetics for the extracellular matrix, or ECM), and pro-regenerative signaling factors. One of the key tissue engineering techniques involves growing cells in three-dimensional (3D) environments, in contrast to the traditional in vitro culture on flat and stiff plastic and glass surfaces. There is a vast body of literature on the advantages of the 3D cell culture over the 2D (traditional) one. However, it has gradually become appreciated that increasing the dimensionality of the cell culture environments from the 2D to the 3D one is not enough. The last decade of research has led to the understanding of the high level of reciprocal interdependency between the cells and their microenvironments, which are unique for every organ, tissue type, functional state, and age. It, therefore, should be considered in every attempt to rebuild the functional or diseased tissue artificially.

This Special Issue provides a snapshot of the state-of-the-art approaches to biologically accurate reconstruction of living structures for regenerative medicine and 3D cell culture. It consists of the works originating from 11 countries around the globe, including four review articles [1,2,3,4] and five reports on the original research [5,6,7,8,9] that were submitted between the 11th of April 2021 and the 25th of March 2022. Below, the key statements of contributing publications are discussed. Next, we propose our summary of the advances, trends, and challenges in the engineered microenvironments for 3D cell culture and regenerative medicine that become visible as a result of collective efforts of the authors of this Special Issue. The overview of the main topics addressed in this Special Issue is shown in Figure 1.

The review papers, contributing to this Special Issue, present an interesting analysis of several leading and emerging concepts, challenges, and methodologies of the field. In particular, Hao and colleagues [1] define the key components of engineered extracellular environments and explore the methods of their optimization for better outcomes in tissue reconstruction. Leslie et al. [2] bring to the light a complex and clinically relevant problem of biologically accurate experimental modelling of the cellular microenvironments that emerge in lungs affected by emphysema. Another crucial aspect of reconstruction of cellular microenvironments for regenerative medicine such as efficient and reliable production of large amounts of cells for transplantation and tissue engineered tissues growth is addressed in a review by Nogueira et al. [3] that discusses the transition of the bioreactor cell culture technologies to the application of disposable processing tanks. In addition to the analysis of putting cells into the specialized microenvironmental contexts, Klontzas and Protonotarios [4] provide a well-structured overview of the current and novel imaging technologies available for monitoring of the functional state of the developing tissue engineering constructs and regenerating organs.

An original idea of the extracellular microenvironments as complex niches that ensure cells’ optimal functionality and tissue reparative potential is presented in the review [1]. This concept complements a conventional setup of the “tissue engineering triad” (cells, scaffolds, and signaling factors) with a more detailed vision of the cellular surroundings and incorporates three principal elements, such as extracellular matrix (ECM), extracellular vehicles (EVs) and growth factors (GFs). Understanding of the effects of each of the elements in their native forms and artificial mimics on cellular activities is essential for the purpose-built design of tissue engineering systems. In the paper, every component of the extracellular microenvironments is considered from three aspects, including native prototypes and their artificial or engineered counterparts, as well as the advantages and limitations of the related materials and technologies.

First, the authors discuss the composition of the native ECM and emphasize the role of the control over a specific integrin type binding and additional functionalization of collagen biomaterials to improve the quality of regenerates. However, the attractive biological properties of the native ECM are not easily translatable into manufacturing, and therefore it remains quite difficult to scale up the production of the reproducible natural ECM-based scaffolds. Artificial biomaterials allow more processing options, including electrospinning and 3D printing, while their modification by using native ECM-derived or ECM-mimicking ligands is indicated as a leading strategy for the creation of the cell-bearing substrates for regenerative medicine and experimental tissue engineering. When analyzing further advantages and limitations of the native ECM biomaterials and artificial ECM-mimetics, the authors pointed out that the dynamic nature of the cell-ECM interactions is still challenging to reproduce by the existing methodologies. The next component of the extracellular microenvironments, according to this study, are exosomes, microvesicles and apoptotic bodies that are produced by the cells and together termed extracellular vesicles (EVs). The main potential of the EVs is linked to their ability to transfer signaling stimuli and deliver therapeutic cargo to optimize the functional status of the cells or modify cell–cell and cell–ECM communications. The promising strategies that allow more efficient application of EVs for regenerative purposes include equipping the vesicles with targeting moieties that would help in their longer retaining in the desired areas, EV-cargo engineering for enhanced loading with therapeutic molecules, and creation of the biomaterials loaded with native or engineered EVs. The authors indicate that the main challenges that need to be addressed are the development of robust methodologies for purification and maintenance of integrity and biological activity of the engineered EVs. Finally, a few strategies to increase the local delivery and retainment of GFs were proposed. For example, the engineered ECMs and scaffolds with increased electronegativity will allow more efficient binding of GFs that contain positively charged amine groups. The main approach selected in the review with regard to the use of the artificial GFs for tissue engineering relies on short biologically active peptide fragments of GFs (GF-mimicking peptides). It is suggested that the efforts in GFs’ usage in regenerative medicine have to be focused on the development of the bioactive GF mimetics with prolonged half-life, lower immunogenicity and higher stability.

Fibroblasts are the main cell type of connective tissue and organs’ stroma. These cells are specialized producers of the structural proteins of ECM such as collagen and elastin, as well as many other ECM molecules, and also the key controllers of matrix remodeling [10,11]. It is well established that fibroblasts are sensitive to the substrate stiffness, and prone to the phenotype change towards the contractile and synthetically active myofibroblasts when in contact with more rigid surfaces [12,13,14,15]. The mechanotransduction cascades involved in these processes are closely associated with the pathological mechanisms of fibrosis [14,15,16]. These principles are widely discussed in the literature.

Surprisingly, the biomechanical aspects of the opposite to fibrosis types of pathology, where the ECM is not excessively deposited, but rather insufficiently maintained or disproportionately resorbed are yet to be studied in detail. Leslie et al. [2] identified a burning question in this area such as lack of knowledge, conceptualization and experimental methodologies for the understanding of the phenomenon of “lazy fibroblasts” observed in the human lung emphysema. During this incurable and common disease, an extensive damage of lung parenchyma is associated with the inability of the resident fibroblasts to repair the ECM and support the unique native mechanical properties of the organ. Authors discuss the existing data on the role of mechanical cues in the control of fibroblasts phenotype and activity, and point out to the fact that the currently used in vitro and in vivo models of lung emphysema are not biologically accurate in terms of the reconstruction of the disease-specific mechanical microenvironment. The authors emphasize this as a burning call for action. According to the referred sources, several available in vitro model systems can be adapted for the modelling of lung emphysema. In particular, it is suggested that the cyclic stretching has to be applied in 2D and 3D models of lung tissue. It is also important to address the soft nature of emphysema tissue, cellular heterogeneity of the lungs, and the role of chronic inflammation to trustfully reconstruct the emphysema tissue in the experiments.

Bioreactors are specialized closed engineered systems for reproduction of various biological processes under strictly controlled conditions [17]. They are essential for the scaling up of preclinical research and clinical translation of tissue engineering advances. The systematics, principles, as well as advantages and limitations of various bioreactor technologies are well addressed in literature [17,18,19,20,21,22,23,24,25,26,27,28,29,30,31,32].

The review by Nogueira et al. [3] explores one of the most important aspects related to the production of cells and cellular products for clinical regenerative medicine applications, such as mutual adaptation of different types of bioreactors and the disposable (single-use) culture chambers (tanks). The need to transition to the disposable systems for bioreactor-based cell culture stems from the regulatory requirements targeting reduction of the risks of contamination of the implantable/injectable products. Importantly, the authors of this article provide a lot of practical information on the commercially available single-use tissue culture bioreactors and their comparisons with the reusable systems.

Klontzas and Protonotarios [4] provide an application-focused analysis of the currently available imaging methodologies for visualization and monitoring of various classes and types of bioartificial tissues, including those cultured extracorporeally and the ones implanted in the human or animal bodies. In addition to the overview of the main principles and areas of potential application of research-grade and clinical-grade imaging techniques, authors summarized the data from published reports regarding the use of the visualization techniques in different organ-specific areas of regenerative medicine and tissue engineering with special attention to cardiovascular, musculoskeletal, and neural tissue engineering.

One of the challenging problems in regenerative medicine is the delivery of signaling factors, genes and drugs to the predefined tissue areas [33]. This potentially can be achieved by using therapeutic-loaded implants [34,35,36,37,38,39,40,41,42,43,44]. However, such implantation may be complicated by the development of foreign body reactions (FBR) that result in the deterioration of the treatment efficacy. One of the common causes of excessive FBR is poor congruency between the implant and surrounding tissues, which results in implant displacement and formation and contraction of the fibrotic peri-implant capsules [45,46,47]. An and Kim [5] approached this challenge through creation of the scaffold systems of organ-specific complex shape. To achieve this, they applied a serial casting method to obtain the detailed replica of the modelled organ and reconstructed the obtained surface profiles using an alginate hydrogel. Notably, the authors, being inspired by mussel biochemistry, modified alginate with catechol. This compound (catechol) has found multiple applications in tissue engineering due to its excellent properties as a wet-resistant adhesive protein, which is compatible with various biomaterials [48]. As a result, it was demonstrated that alginate–catechol hydrogel can be used to create complex-shaped 2D films and 3D structures. Moreover, it was highly biocompatible and allowed the delivery of large amounts of therapeutic cargo. This study shows an inspiring example of engineering of a scaffold-based implantable drug delivery system, but also provides a methodology for the reconstruction of the organ- and tissue-specific geometries of high complexity.

Several studies in the current Special Issue are exploring the conditions and principles of engineering of organ- and disease-specific cellular microenvironments.

Reconstruction of the bone tissue in in vitro conditions is a difficult challenge. The osteocytes are extremely sensitive to the varying mechanical loads in the bone and adapt its structure by intensive remodeling of ECM. One of the main and unique aspects of this process is dynamic biomineralization. The fact that the osteocytes are embedded in the hard mineralized matter with complex 3D geometry is largely ignored in the conventional in vitro bone models, as the cells are grown on stiff and flat 2D substrates. Brady, O’Brien and Hoey [9] have demonstrated that the linear bone cells that are commonly used in bone research in vitro (MLO-Y4), under standard 2D culture conditions, lose their ability to express the *Sost* gene. This gene codes a protein sclerostin, which plays a pivotal role in regulation of the adaptive bone mineralization [49,50] and currently considered as a promising therapeutic target for the treatment of various disorders of bone tissue integrity, including osteoporosis [51]. The authors of the study [9] have proved that *Sost* gene expression in MLO-Y4 cells can be rescued by controlled changes of the cellular microenvironment. They found that on hydroxyapatite-enriched 2D film, the cells start to re-express this gene, while when cultured on 3D collagen-hydroxyapatite composite scaffolds, MLO-Y4 line becomes capable of producing sclerostin at physiological levels. This work makes an important contribution to bone research by creating a feasible methodology that allows culturing an accessible and standard osteocyte cell line under conditions where these cells demonstrate biologically relevant behavior. The outcomes of the study may be applicable to explore the mechanisms of bone remodeling and to develop new treatment for bone diseases.

The work of Soleas et al. [8] shows that the geometry of the substrate as a physical cue controls the differentiation of lung progenitor cells derived from embryonic stem cells. Using tubular PDMS substrates with the diameters mimicking different parts of the developing embryonic lungs, these researchers recently identified the diameter of the tube culture substrate as a necessary factor to induce the site-specific cellular phenotypes of the lung epithelial cells [52]. In the current work, this group demonstrated that the same physical cue controls the gene expression profile of the lung progenitor cells, while this effect is independent on the ECM coating of the tube surface and initial seeding density. Authors revealed the effects of the tube diameter on the morphology of the cells, and their ability to self-assemble into a stable monolayer lining of the internal surface of the mold. It is suggested that the insensitiveness of the self-assembling of the monolayer to the ECM composition can be explained by possible secretion of the own matrix by the cells themselves as soon as they adhered to the tube walls. Additionally, following the analysis of the experimental conditions, the dominance of the morphogenetic significance soluble factors over the bounded ligands can be proposed. The take-home message of this study is that the physical cues can be used to augment chemically induced embryonic stem cell differentiation. This methodology can potentially be translated and scaled up for the development of other types of tubular structures and organs, and better reproducibility of the organoids’ 3D cell culture. Importantly, in this work, an uncommon controlled combined application of chemical and physical stimuli was demonstrated in the creation of a complex organ-specific engineered microenvironment.

Vascularization of the reconstructed tissues is a long-standing and critical challenge on the way to the efficient application of tissue engineering technologies and constructs for the treatment of chronic wounds. Such lesions have insufficient blood supply and therefore they are highly hypoxic. In these conditions, the grafted tissues, cells and tissue engineering constructs demonstrate limited survival and regeneration potential [53,54]. It is well recognized that the strategies allowing creation of stable prevascularized engineered grafts provide the most promising answers to this challenge. In the article presented by Duan and colleagues [7], a thorough approach for the optimization of the microenvironment for the development of vascular networks in hydrogel substrates in vitro has been demonstrated. An elegant experiment was performed to identify the specific contributions of fibronectin and vascular smooth muscle cells (VSMC) derived from the human-induced pluripotent stem cells to the angiogenic response of endothelial cells. Collagen hydrogels were modified with fibronectin and seeded with either endothelial cells only or a mixture of VSMC and endothelial cells. It was found that fibronectin enhanced angiogenesis (the effect was mediated through the integrin αvβ3), while VSMC further augmented the quality of the vascular networks (especially their number and size) via the secretion of vascular endothelial growth factor and basic fibroblast growth factor. This work makes a critical step for the development of reproducible pre-vascularized hydrogels for wound healing by combining ethically sourced cells and accessible extracellular matrix components.

Liver fibrosis is the most common complication of hepatic acute and chronic inflammation and traumas and a major source of global morbidity and mortality. The key feature of liver fibrosis is the excessive accumulation of ECM and transformation of the functional organ parenchyma into a kind of scar tissue [55]. The progress of liver fibrosis is therefore associated with the increasing stiffness of the tissue. However, so far, only a few experimental models have revealed the attempts to simulate and monitor these changes of mechanical properties in real time in 3D in vitro conditions.

Cacopardo and Ahluwalia [6] presented a new rational and feasible methodology to model time-evolving mechanical properties of the liver tissue affected by fibrosis. Moreover, their approach that involved the usage of customized micromechanical bioreactor, allowed precise and non-destructive measure the tissue modifications. The authors demonstrated a 2-step protocol of increasing the stiffness of the gelatin hydrogels embedded with HepG2 hepatocyte-like cells by sequential crosslinking of the gels with microbial transglutaminase. In contrast to the previously reported models that involved photo-crosslinking-based techniques, the application of transglutaminase was found to be non-cytotoxic. It is worth to be emphasized that this study represents, at least, one of the first if not the unique, successful attempt to reproduce the dynamic changes of ECM during the disease progression in parallel with the biological assays and mechanical monitoring of the state of the obtained soft tissue engineering construct.

In conclusion, this Special Issue has revealed a few areas of focused attention in the global tissue engineering and regenerative medicine research community. The importance of combining several factors to create biologically relevant engineered microenvironments for cell culture has been emphasized. Some of such factors have been highlighted, including the organ- and disease specificity, the capability for dynamic remodeling and adaptation, and the crucial role of physical cues in governing the cells’ fate. Notably, the emerging geometry parameters such as the dimensionality and linear dimensions of the living space of the cells, the confinement, the surface curvature, and shape congruency have been explored in the studies contributing to this Special Issue. In addition, the importance of the adaptable and coordinated development of the instruments to support the proposed advanced methods was stressed, including the bioreactor technologies and imaging modalities. We believe that the most significant challenge that can be derived from the analysis of the publications of this issue is the limited availability of the approaches that would allow the creation of the engineered microenvironments for the 3D culture and regenerative medicine with more than one controlled parameter.

## Figures and Tables

**Figure 1:**
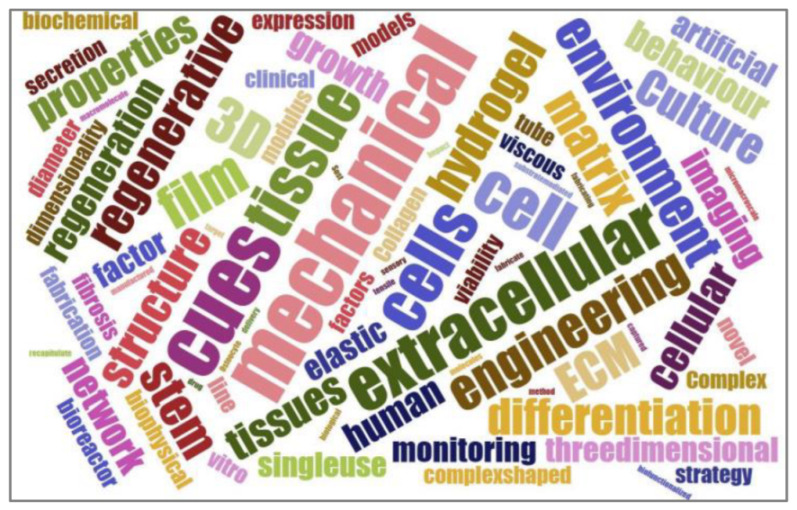
Word cloud reflecting the contents of this Special Issue.

## Data Availability

Data is contained within the article.
